# Potentiation of radiation therapy by the oncolytic adenovirus dl1520 (ONYX-015) in human malignant glioma xenografts

**DOI:** 10.1038/sj.bjc.6601102

**Published:** 2003-07-29

**Authors:** B Geoerger, J Grill, P Opolon, J Morizet, G Aubert, Y Lecluse, V W van Beusechem, W R Gerritsen, D H Kirn, G Vassal

**Affiliations:** 1 Department of Pediatrics, Institut Gustave-Roussy, 94805 Villejuif, France; 2 Pharmacology and New Treatments of Cancers (UPRES EA3535), Institut Gustave-Roussy, 94805 Villejuif, France; 3 Vectorology and Gene transfer (UMR8121), Institut Gustave-Roussy, 94805 Villejuif, France; 4 Flowcytometry Facility, Institut Gustave-Roussy, 94805 Villejuif, France; 5 Division of Gene Therapy, Department of Medical Oncology, Vrije Universiteit Medical Center, 1007 MB Amsterdam, The Netherlands; 6 ONYX Pharmaceuticals, Richmond, CA 94806, USA

**Keywords:** adenoviral cytolysis, radiosensitization, brain tumour, xenografts

## Abstract

In spite of aggressive surgery, irradiation and/or chemotherapy, treatment of malignant gliomas remains a major challenge in adults and children due to high treatment failure. We have demonstrated significant cell lysis and antitumour activity of the *E1B*-55 kDa-gene-deleted adenovirus ONYX-015 (dl1520, CI-1042; ONYX Pharmaceuticals) in subcutaneous human malignant glioma xenografts deriving from primary tumours. Here, we show the combined efficacy of this oncolytic therapy with radiation therapy. Total body irradiation (5 Gy) of athymic nude mice prior to intratumoral injections of ONYX-015 1 × 10^8^ PFU daily for 5 consecutive days yielded additive tumour growth delays in the *p53* mutant xenograft IGRG88. Radiation therapy was potentiated in the p53 functional tumour IGRG121 with a ‘subtherapeutic’ dose of 1 × 10^7^ PFU daily for 5 consecutive days, inducing significant tumour growth delay, 90% tumour regression and 50% tumour-free survivors 4 months after treatment. These potentiating effects were not due to increased adenoviral infectivity or replication. Furthermore, cell lysis and induction of apoptosis, the major mechanisms for adenoviral antitumour activity, did not play a major role in the combined treatment strategy. Interestingly, the oncolytic adenovirus seemed to accelerate radiation-induced tumour fibrosis. Potentiating antitumour activity suggests the development of this combined treatment for these highly malignant tumours.

Anaplastic astrocytoma and glioblastoma, the most common primary brain tumours in adults and childhood, are quite refractory to current treatment with median survival rarely exceeding 1 year (Holland, 2000). Current first-line treatment for malignant gliomas is surgery and radiation therapy and/or chemotherapy. However, *p53* mutations, mdm2 amplification/overexpression, or deletions of the *CDKN2A/p14^ARF^* tumour-suppressor gene often render the gliomas resistant to these therapeutic approaches (Frankel *et al*, 1992; Van Meir *et al*, 1994; Iwadate *et al*, 1996). Replication-selective oncolytic adenoviruses that can replicate in and cause lysis of tumour cells, but spare normal cells, have been introduced recently as new therapeutic strategies and are currently evaluated in clinical trials. We have demonstrated significant antitumour activity of the *E1B* 55 kDa-gene-deleted, replicative adenovirus ONYX-015 (dl1520, CI-1042; ONYX Pharmaceuticals) in human malignant glioma tumour xenografts derived from primary tumours (Geoerger *et al*, 2002). Although initially suggested to replicate selectively in *p53* mutant tumour cells, ONYX-015-mediated cytolysis in our tumour xenografts was irrespective of cellular p53 status.

Several reports have shown that ONYX-015 is most effective when combined with conventional anticancer therapies. In this regard, ONYX-015 has been described to increase chemosensitivity *in vitro* and in preclinical *in vivo* xenograft models (Heise *et al*, 1997; 2000; Vollmer *et al*, 1999; You *et al*, 2000). Clinical phase II trials combining intratumoral ONYX-015 injection with chemotherapy cisplatin and 5-fluorouracil in patients with recurrent head and neck cancer has shown promising results (Khuri *et al*, 2000). Furthermore, ONYX-015 has been suggested as an effective neoadjuvant to radiation therapy in a human colon carcinoma model (Rogulski *et al*, 2000). Radiation therapy plays the central role in the current treatment of malignant gliomas. We therefore investigated the possible radiosensitising effect of ONYX-015 on malignant glioma cells. The present report provides evidence that ONYX-015 has an additive, even potentiating antitumour effect on irradiated human brain tumour xenografts supporting the use of combined treatment with this attenuated replicative adenovirus and radiation therapy for malignant gliomas.

## MATERIALS AND METHODS

### Adenovirus

The chimeric human group C adenovirus ONYX-015 contains a deletion between nucleotides 2496 and 3323 in the *E1B* region encoding the 55 kDa protein and a C → T transition at position 2022 in *E1B* generating a stop codon at the third codon position of the protein (Barker and Berk, 1987). These alterations eliminate expression of the *E1B* 55 kDa gene in ONYX-015-infected cells. The virus was generously provided by ONYX Pharmaceuticals (Richmond, CA, USA).

### Xenografts and experimental design

Antitumour activity was evaluated against advanced stage human malignant glioma **IGRG88** (*p53* mutant) and glioblastoma xenograft **IGRG121** (*p53* wild-type) derived from primary tumours, previously described (Geoerger *et al*, 2002). All animal experiments were carried out with ethical committee approval and under the conditions established by the European Community (Directive 86/609/CCE) and in accordance with the UKCCCR guidelines (Workman *et al*, 1998). Female SPF-Swiss athymic nude mice bearing s.c. tumours of 100–300 mm^3^ were randomly assigned to treatment groups on day 0 of treatment. Tumours were measured three times weekly and tumour volume calculated according to the equation: *V* (mm^3^)=width^2^ (mm^2^) × length (mm)/2. The experiment was stopped after 120 days when there were tumour-free survivors. Statistical significance between treatment groups and controls was estimated by the two-tailed nonparametric Kruskal–Wallis test. Tumour regression was described using standard terminology. ‘Additive’ antitumour activity was defined as an antitumour effect of the sum of both agents alone, ‘synergy’ as effects greater than the sum of both agents alone, and ‘potentiation’ as the effect of an itself nontoxic agent to increase the efficacy of the combined agent.

### Radiation and adenoviral treatment

X-rays were delivered under a tension of 225 kV and 17 mA, using a filter of 0.5 mmCU with an RT250 Phillips. Total body irradiation was performed once on day 0 at a dose of 5 Gy (maximum tolerated dose). Following irradiation, animals were placed in a system with constant air renewal allowing to perform experiments under L2 conditions. For combined treatment studies, animals bearing IGRG88 xenografts received 5 Gy TBI and intratumoral injections of 1 × 10^8^ PFU ONYX-015 in 50 *μ*l PBS for 5 consecutive days. Animals bearing IGRG121 xenografts, received TBI and a dose of 1 × 10^7^ PFU ONYX-015 for 5 days. The first virus injection was performed 6–7 h following irradiation in anaesthetized animals. Different sites of the tumour were chosen for each injection. Simultaneously, groups of animals were either irradiated with 5 Gy and injected with vehicle PBS without glycerol (Gibco Invitrogen SARL, Cergy Pontoise, France) for 5 days, or were enclosed in a radiation chamber without radiation and received injections of 1 × 10^8^ PFU ONYX-015 or 1 × 10^7^ PFU ONYX-015, respectively, for 5 days, or control animals were enclosed in the radiation chamber without radiation and were injected with PBS in an equivalent volume and schedule. Experiments were carried out with ethical committee approval and under the conditions established by the European Community (Directive 86/609/CCE) and in accordance with the UKCCCR guidelines (Workman *et al*, 1998).

### Infectivity using Ad-luciferase

Short-term cultures of IGRG88 and IGRG121 glioma cells (10^4^/well in a 96-well plate) in triplicate were irradiated with 0, 2, and 5 Gy and incubated 48 h later with AdCMVLuc at 10 or 100 MOI. Luciferase activity in the cells was assayed 48 h after infection using the Luciferase Assay System (Promega, Madison, WI, USA) and a Lumat LB 9507 luminometer (EG&G Berthold, Bad Wildbad, Germany). Infectivity of glioma cells was defined by the amount of luciferase activity measured after adenoviral-mediated gene transfer in the tumour cells.

### Flow cytometry for cell cycle

Subcutaneous IGRG88 and IGRG121 tumours were harvested at baseline and 7, 24, and 48 h after 5 Gy TBI irradiation treatment and were frozen at −80°C. Tumour cells were aspirated from frozen tumours with a 25 Gauge needle and resuspended to approximately 3 × 10^6^ cell ml^−1^ in Tris buffer, pH 7.6 (100 mM Trizma® Base, 82 mM HCl, 100 mM NaCl) without further enzymatic cell-separation step. Cells were stained with propidium iodide buffer (10 mM NaCl, 3.4 mm trisodium citrate, 0.001% propidium iodide, 0.1% Nonidet P-40, and 0.005% RNase) for 1 h. The DNA content of individual cells was measured using a FACScalibat (Becton Dickinson).

### Adenovirus cytopathic effect assay

IGRG121 tumour xenografts were injected *in vivo* once with 10^7^ PFU ONYX-015 or 10^8^ PFU ONYX-015, or 10^7^ PFU ONYX-015, 7 h after 5 Gy TBI. Experiments were carried out with ethical committee approval and under the conditions established by the European Community (Directive 86/609/CCE) and in accordance with the UKCCCR guidelines (Workman *et al*, 1998). Tumours were harvested 1, 3, and 6 days after treatment and stored at −80°C. Tumours were grinded on fluid nitrogen and resuspended in PBS. Virus was released from cells by freeze/thaw cycles. Serial dilutions on the lysates were titred on the human retinoblast cell line 911 for cytopathic effect *in vitro*. Briefly, 10^4^ 911 cells per well were plated in DMEM supplemented with 10% fetal bovine serum (Gibco Invitrogen SARL, Cergy Pontoise, France) in 96-well plates and infected after 24 h with the virus solution at end point dilutions according to standard procedures. Plates were incubated in humidified conditions at 37°C and monitored daily for cytopathic effect during 14 days. Each sample was done in triplicate. Uninfected wells were used as a control for cell survival.

### Histology and immunohistochemistry for adenoviral hexon protein

Formalin-fixed paraffin-embedded tumours, cut into 4-*μ*m thick sections, and rehydrated, were incubated for 1 h at 35°C with the polyclonal antibody AB 1056 (Chemicon International, Temecula, CA, USA) diluted 1 : 300. Detection was performed by a biotinylated rabbit secondary antibody to goat immunoglobulin streptavidin–horseradish peroxidase conjugate (DAKO, Glostrup, Denmark), and the chromogen diaminobenzidine. Slides were counterstained with haematoxylin. Haematoxylin–eosin–safranin staining was performed for morphology; Masson's trichrome staining for collagen type I, was performed according to the manufacturer's instructions and using aniline blue (Reactifs RAL, Martillac, France).

### Terminal deoxynucleotidyl transferase-mediated dUTP nick end labelling (TUNEL)

Terminal deoxynucleotidyl transferase-mediated dUTP nick end labelling (TUNEL) was performed to quantify apoptotic cell death using the *In situ* Cell Death Detection Kit, AP (Roche Diagnostics GmbH, Mannheim, Germany) according to the manufacturer's instructions. Tissue sections were deparaffinised, rehydrated, and received microwave pretreatment in citric acid buffer for antigen retrieval. DNA strand breaks were labelled by polymerisation of fluorescein-dUTP to the 3′-OH sites, catalysed by terminal deoxynucleotidyl transferase (TdT), revealed by antifluorescein Fab fragments conjugated with alkaline phosphatase (Converter-AP), and visualised using Fast Red (DAKO, Glostrup, Denmark). TdT and dUTP were omitted for negative controls. TUNEL-positive cells, displaying compaction or segregation of the nuclear chromatin, or breaking up of the nucleus into discrete fragments, were counted per view at × 100 magnification. Five representative fields were chosen for counting; necrotic fields were excluded.

## RESULTS

### Radiation therapy and ONYX-015 have potentiating antitumour activity in human malignant glioma xenografts *in vivo*

Antitumour activity of ONYX-015 in combination with radiation therapy *in vivo* was evaluated in the moderately radiosensitive human malignant glioma xenografts IGRG121 and IGRG88 deriving from primary tumours. To prevent potential mutagenesis of adenoviral DNA, irradiation was delivered prior to intratumoral injections of ONYX-015. Intratumoral administration of ONYX-015 at 1 × 10^8^ PFU/injection on 5 consecutive days to mice bearing advanced stage subcutaneous **IGRG88** tumour xenografts resulted in significant tumour growth delay of 10 days compared to controls (*P*<0.05) and one and two out of 10 animals experienced complete and partial tumour regression, respectively (Table 1

**Table 1:** Antitumour activity of ONYX-015 (intratumoral injection) and radiation therapy in subcutaneous malignant glioma xenografts

	**Treatment intratumoral**	***n***	**PR^a^**	**CR^b^**	**TFS^c^**	**5 × vol days**	**TGD^d^ days**	***P*^e^**
*IGRG88 (mutant p53)*								
Control	PBS	10	0	0	0	12	–	
Rx	5 Gy^f^+PBS	11	0	0	0	27	15	<0.01
ONYX-015	10^8^ PFU day^−1^ × 5	10	2	1	1	22	10	<0.05
ONYX-015+Rx	5 Gy^f^+10^8^ PFU day^−1^ × 5	11	8	0	0	42	30	<0.001
								
*IGRG121 (wild-type p53)*								
Control	PBS	9	0	0	0	6	–	
Rx	5 Gy^f^+PBS	9	0	0	0	17	11	<0.01
ONYX-015	10^7^ PFU day^−1^ × 5	10	0	0	0	11	5	NS
ONYX-015+Rx	5 Gy^f^+10^7^ PFU day^−1^ × 5	10	4	5	5	81	75	<0.001

a Partial regression;

b complete regression;

c tumour-free survival;

d tumour growth delay;

e for each experiment, the 5 × volumes in the treatment groups were compared to those of the control group using a two-tailed nonparametric Kruskal–Wallis test;

f total body irradiation.

, Figure 1A

**Figure 1:**
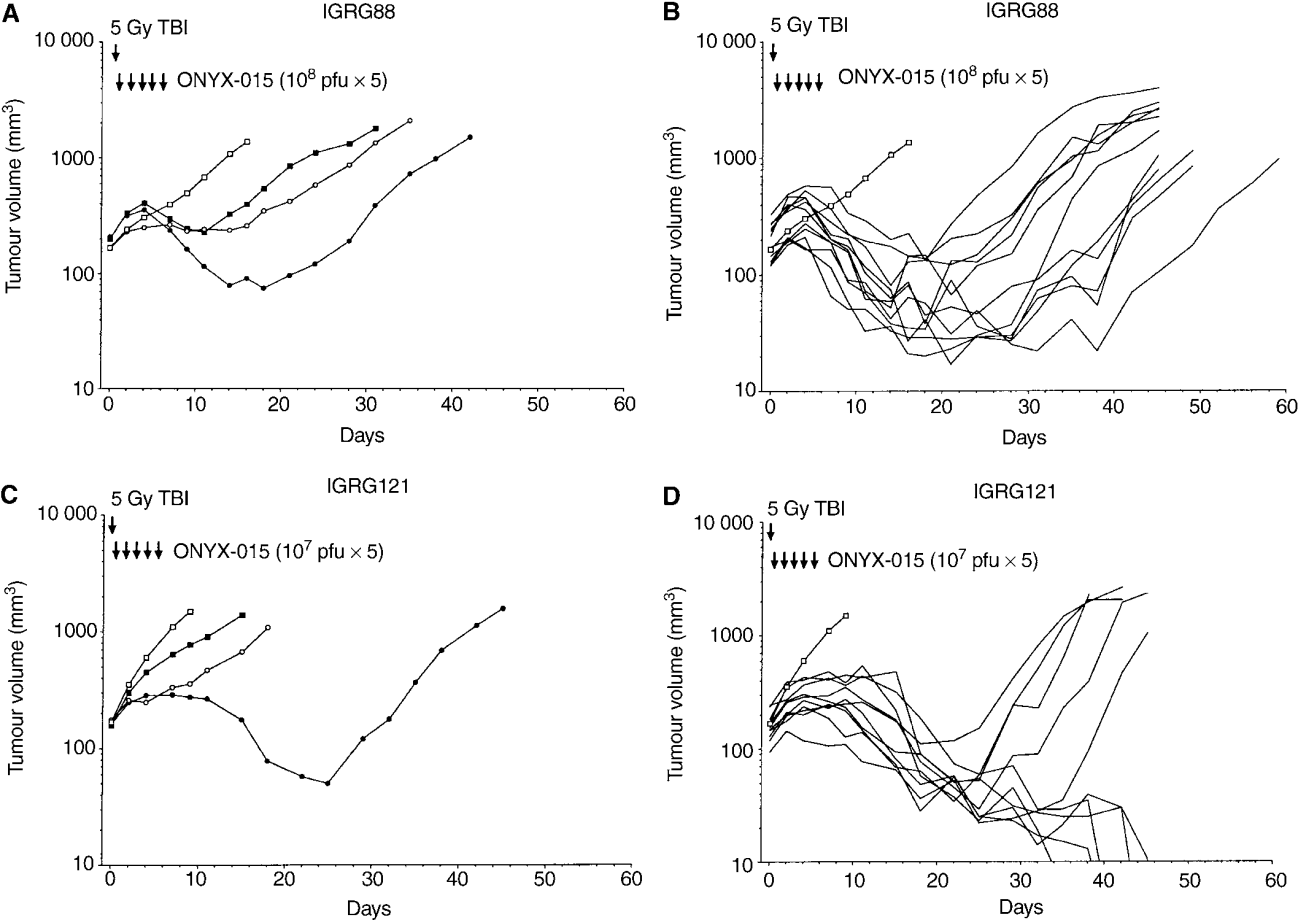
Antitumour activity of radiation therapy (5 Gy TBI) and ONYX-015 in *p53* mutant IGRG88 and *p53* wildtype IGRG121 subcutaneous malignant glioma xenografts. Mice bearing subcutaneous IGRG88 (**A**, **B**) and IGRG121 xenografts (**C**, **D**) were randomly assigned to four groups: control animals injected with PBS (open square □), animals irradiated with 5 Gy TBI (open circle ○), animals injected with ONYX-015 10^8^ PFU day^−1^ × 5 in IGRG88, and 10^7^ PFU day^−1^ × 5 in IGRG121 (solid square ▪), and animals irradiated with 5 Gy TBI and injected with ONYX-015 10^8^ PFU day^−1^ × 5 or 10^7^ PFU day^−1^ × 5, respectively (solid circle •). Panels (**A**) and (**C**) give the means of all treatment groups, panels (**B**) and (**D**) show the individual tumours of animals subjected to combination treatment compared to the mean of the controls. Each line represents one individual tumour.

). Total body irradiation of 5 Gy induced a significant tumour growth delay of 15 days compared to untreated controls (*P*< 0.01). Moreover, combined treatment of irradiation given 6–7 h prior to the first intratumoral ONYX-015 treatment yielded an additive antitumour activity with 73% partial tumour regressions and TGD of 30 days (*P*<0.001, compared to untreated controls) (Table 1, Figure 1A and B).

In the **IGRG121** xenografts, intratumoral treatment with ONYX-015 at 1 × 10^8^ PFU times 5 has previously been proven highly effective, inducing 73% tumour-free survivors at 4 months (Geoerger *et al*, 2002). To be able to demonstrate additive or even synergistic effects, the study in this xenograft was conducted with a decreased dose of 1 × 10^7^ PFU daily ONYX-015 injections. At this dosage, ONYX-015 induced neither significant tumour growth delay nor tumour regression (Table 1; Figure 1C). Total body irradiation induced a significant tumour growth delay of 11 days compared to controls (*P*<0.01). Most importantly, the ‘subtherapeutic’ dose of ONYX-015 potentiated antitumour activity of total body irradiation and induced a significant tumour growth delay of 75 days compared to untreated controls (*P*<0.001) and 40% partial and 50% complete tumour regressions with five of 10 animals surviving tumour-free 120 days after treatment (Table 1; Figure 1C and D).

### Irradiation does not increase adenoviral infectivity in malignant glioma cells *in vitro*

**Figure 2:**
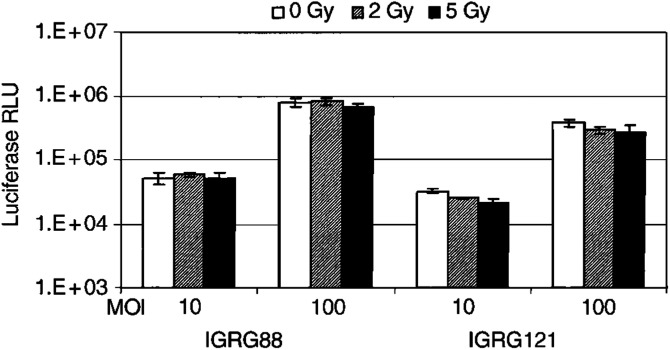
*In vitro* infectivity of IGRG88 and IGRG121 glioma cells 48 h after irradiation with 2 and 5 Gy using AdCMV Luciferase. IGRG88 and IGRG121 glioma cells in short-term cultures were irradiated with 2 Gy (hatched bars) and 5 Gy (black bars), and infected with the AdCMVLuc virus at an MOI of 10 and 100. White bars represent nonirradiated controls. Luciferase activity values represent means of triplicate; error bars mark the standard deviations.

The coxsackie adenoviral receptor (CAR) plays a crucial role in the viral infection of cells, with *α*v integrins promoting internalisation. Previously, we have shown expression of CAR in the malignant glioma tumour IGRG88, but hardly any expression in the highly ONYX-015-sensitive tumour IGRG121 (Geoerger *et al*, 2002). To investigate whether irradiation might sensitise tumour cells to adenoviral infection through upregulation of receptor expression, we determined expression of CAR and *α*v integrins after RT in CAR negative glioma cells *in vitro*. However, expression of both receptors was not significantly influenced through prior radiation treatment (data not shown). In addition, we examined whether irradiation influences viral infectivity of glioma tumour cells using an adenovirus vector expressing luciferase under the CMV promoter. Transgene expression of the IGRG88 and IGRG121 glioma cells *in vitro*, infected at 10 and 100 MOI, was not increased 48 h after irradiation with 2 or 5 Gy compared to untreated controls (Figure 2

). These observations were further confirmed in glioma cell lines (U118-MG, U373-MG, Gli-6; data not shown). In fact, infectivity of IGRG121 was not superior to that of IGRG88, suggesting that the efficacy *in vivo* was not determined by infection efficiency alone.

### Radiation therapy does not significantly increase S-phase fraction, but induces cell cycle arrest

**Figure 3:**
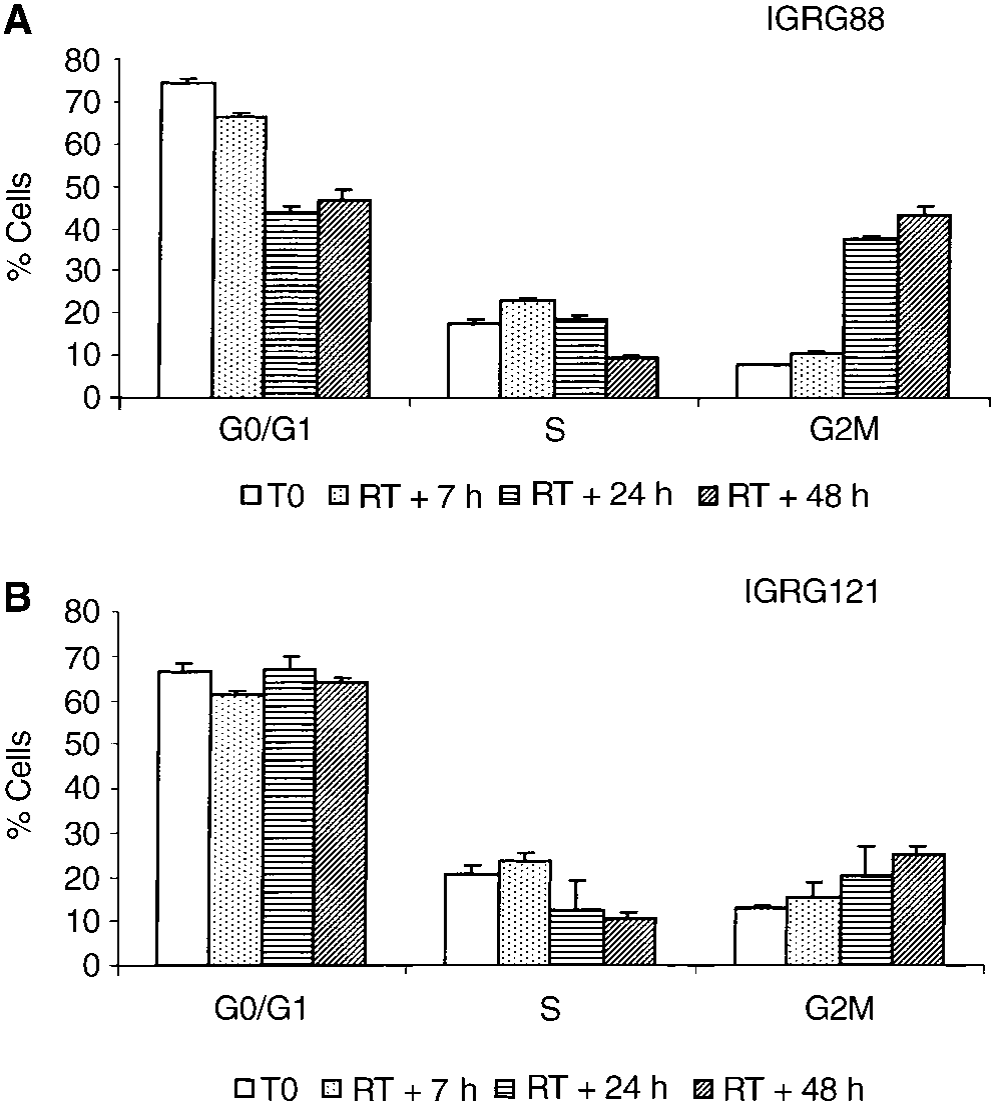
Cell cycle status in IGRG88 (**A**) and IGRG121 (**B**) tumour cells following irradiation treatment. Subcutaneous IGRG88 and IGRG121 tumours were harvested at baseline (white bars) and 7 h (dotted bars), 24 h (horizontal hatched bars), and 48 h (hatched bars) after 5 Gy TBI irradiation treatment. Aspirated tumour cells were stained with propidium iodide buffer and DNA content was measured using flow cytometry. Values represent means of triplicate; error bars mark the standard deviations.

Cellular S-phase fraction has been suggested to determine sensitivity of cells to viral infection (Goodrum and Ornelles, 1998). To investigate if irradiation facilitates viral infection and/or replication through a shift of tumour cells into S phase, we analysed cell cycle status of the subcutaneous IGRG88 and IGRG121 tumours by FACS at baseline and 7, 24, and 48 h after 5 Gy TBI (Figure 3

). As expected, IGRG121 with functional p53 arrested in G0/1 and in G2 M during at least 48 h after irradiation. *P53* mutant IGRG88 cells arrested only in G2 M and not in G0/1. However, there was only a minor increase in tumour cells in S phase of 3% (IGRG121) and 5% (IGRG88) after 7 h, with a subsequent drop to 50% of the initial S fraction. These minor and transient changes might not be considered significant enough to explain the potentiating effect of combination treatment *in vivo*.

### Radiation therapy does not increase adenoviral replication in IGRG121 *in vivo*

**Figure 4:**
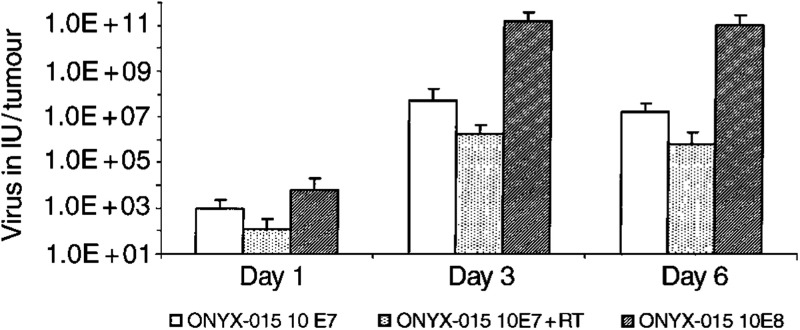
Adenoviral replication in glioblastoma IGRG121 following RT and ONYX-015 *in vivo*. IGRG121 tumours were injected *in vivo* once with 10^7^ PFU ONYX-015 (white bars), 10^8^ PFU ONYX-015 (hatched bars), or 10^7^ PFU ONYX-015 7 h after 5 Gy TBI (dotted bars) and harvested on days 1, 3, and 6. Serial dilutions of tumour cell lysates were titred on the human retinoblast cell line 911 for cytopathic effect. Values represent means of four or five independent tumours each measured in triplicate; error bars mark the standard deviations.

We next examined whether irradiation increases adenoviral replication in IGRG121 tumours *in vivo*. Viral titres of ONYX-015 in tumours injected once with 10^7^ or 10^8^ PFU ONYX-015 were increased by 5 and 7 log, respectively, at days 3 and 6 compared to day 1 after injection, demonstrating significant intratumoral adenoviral replication *in vivo* (Figure 4

). Viral yields in tumours were higher following intratumoral injection of 10^8^ PFU ONYX-015 compared to 10^7^ PFU ONYX-015. Nevertheless, virus titres were not increased through prior radiation treatment in tumours injected with 10^7^ PFU compared to those treated with adenovirus alone. In fact, they were slightly lower.

**Figure 5:**
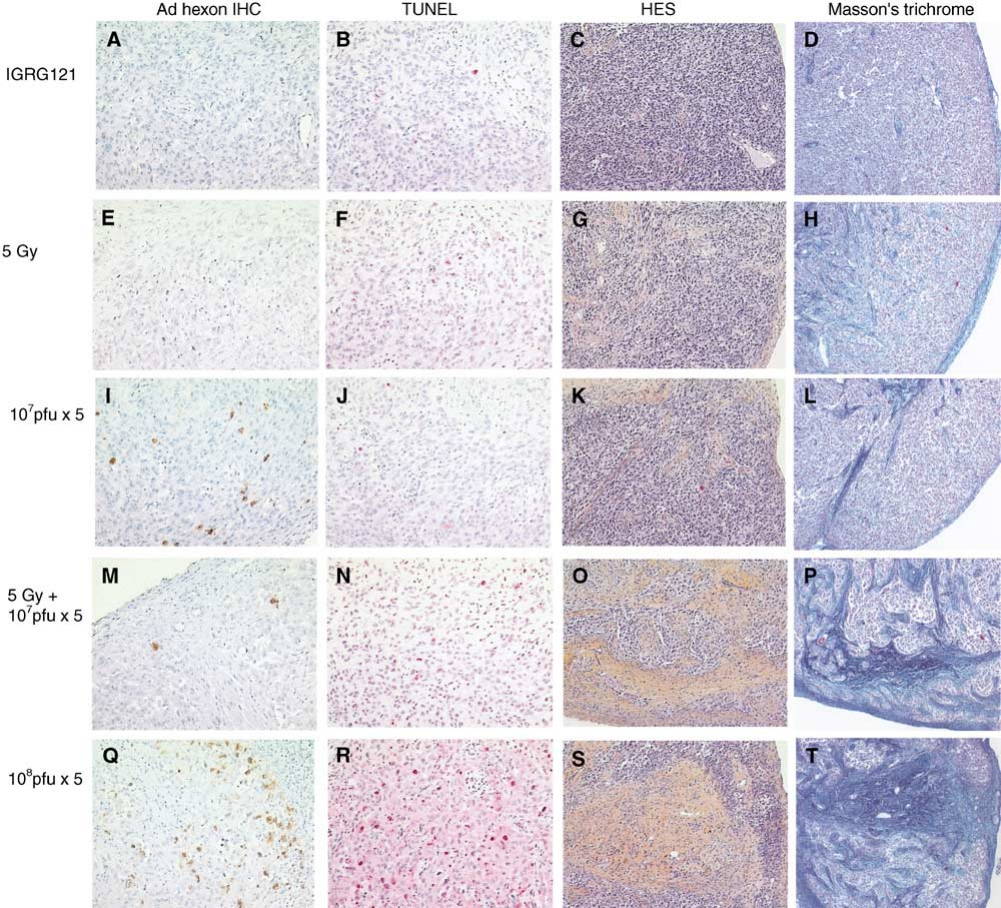
Histology of glioblastoma IGRG121 treated with RT and ONYX-015. Immunohistochemical staining of adenoviral hexon protein (**A**, **E**, **I**, **M**, **Q**), terminal deoxynucleotidyl transferase-mediated fluorescein-dUTP nick end labelling (TUNEL) (**B**, **F**, **J**, **N**, **R**), haematoxilin–eosin–safranin staining (**C**, **G**, **K**, **O**, **S**), and Masson's trichrome staining in IGRG121 human glioblastoma xenografts treated with PBS (**A**, **B**, **C**, **D**), 5 Gy TBI (**E**, **F**, **G**, **H**), ONYX-015 10^7^ PFU day^−1^ × 5 (**I**, **J**, **K**, **L**), 5 Gy TBI and ONYX-015 10^7^ PFU day^−1^ × 5 (**M**, **N**, **O**, **P**) or ONYX-015 10^8^ PFU day^−1^ × 5 (**Q**, **R**, **S**, **T**). Tumours were excised from nude mice on day 5 (IHC and TUNEL staining) or day 10 (HES and Masson's trichrome staining) after treatment start. In IHC, nuclei containing the adenoviral hexon protein are stained brown. Adenoviral replication was not increased after RT (M) compared with ONYX-015 10^7^ PFU day^−1^ × 5 alone (**I**). Five 10^8^ PFU ONYX-015 injections induced significant features of apoptotic cell death displaying compaction or segregation of the nuclear chromatin, or breaking up of the nucleus into discrete fragments ((**R**), TUNEL-positive cells are stained in red) compared to untreated controls (**B**). Apoptotic rate was insignificantly increased 5 days after irradiation treatment of 5 Gy (**F**), ONYX-015 10^7^ PFU day^−1^ × 5 (**J**), and in tumours treated combined with irradiation and ONYX-015 10^7^ PFU day^−1^ × 5 (**N**). ONYX-015 10^8^ PFU day^−1^ × 5 induced preapoptotic changes and tumour cell necrosis (**S**). After 5 Gy TBI and ONYX-015 10^7^ PFU day^−1^ × 5 cells, foci of viable tumour cells were surrounded by inflammatory cells and prominent fibrosis (**O**); collagen type I as marker for fibrosis is stained in blue (**P**). Hexon IHC and TUNEL were photographed at × 100 original magnification, HES and Masson's trichrome staining at 50 × original magnification.

We further performed histology and immunohistochemistry for adenoviral hexon protein on paraffin-embedded tissue sections of IGRG121 tumour xenografts treated with 5 Gy TBI and/or five adenoviral injections. IHC confirmed the findings of viral yields and showed no increase of adenoviral replication at day 5 (Figure 5M

) and day 10 (not shown) in tumours treated with radiation prior to five times 10^7^ PFU ONYX-015 injections compared to ONYX-015 alone (Figure 5I). With or without prior irradiation, adenoviral replication was significantly lower than in tumours injected with the 10-times higher dose of 10^8^ PFU ONYX-015 for 5days (Figure 5Q).

### Induction of apoptotic cell death is not responsible for the potentiating effects of ONYX-015 with RT in IGRG121 *in vivo*

Since the tumour-suppressor protein p53 mediates cell-cycle arrest and/or apoptosis in response to oncogenic signals such as E1A and DNA damage, we determined whether apoptotic cell death might be a major event in the cytotoxic effect of adenovirus ONYX-015 and in combination treatment with RT. Staining on the tissue sections of injected xenografts of the p53 functional IGRG121 at day 5 of ONYX-015 10^8^ PFU × 5 treatment revealed major preapoptotic and apoptotic changes in 3.1% of tumour cells, displaying compaction or segregation of the nuclear chromatin, or breaking up of the nucleus into discrete fragments (Figure 5R). In contrast, no significant increase in apoptotic figures compared to PBS-injected controls (0.21% TUNEL-positive tumour cells; Figure 5B) could be determined in irradiated IGRG121 tumours (0.34% TUNEL-positive tumour cells; Figure 5F), or when treated with the noneffective, but radiation-potentiating dose of ONYX-015 10^7^ PFU × 5 (0.39% TUNEL-positive tumour cells; Figure 5J), or the combination of this adenovirus dose with irradiation (0.42% TUNEL-positive tumour cells; Figure 5N).

### ONYX-015 accelerates radiation-induced tumour fibrosis *in vivo*

HES staining for morphology revealed an interesting aspect of the treated xenografts (Figure 5). IGRG121 tumours treated with the cytotoxic dose of 5 × 10^8^ PFU ONYX-015 showed at day 10 post-therapeutic changes, including few viable tumour cells, with preapoptotic changes and single cell necrosis, surrounded by stromal fibrosis (Figure 5S and T). IGRG121 tumours treated with combined radiation therapy and 10^7^ PFU ONYX-015 × 5 at day 10 revealed few mitotic figures and dystrophic tumour cells (Figure 5O). Tumour cells in many areas were replaced by stromal infiltration; small foci of viable tumour cells were surrounded by fibrosis and inflammatory cells, mainly macrophages and lymphocytes. Masson's trichrome staining determined collagen type I, stained in blue, as the predominant morphological component of these fibrotic changes (Figure 5P). Thus, although treatment with RT and a 10 times lower dose of ONYX-015 induced an antitumour efficacy comparable to that of the higher virus dose alone (Geoerger *et al*, 2002), the cytolytic changes seen after high-dose adenoviral treatment were replaced by major fibrotic changes which were much more profound and occurred more rapidly than in tumours treated with irradiation alone (Figure 5G and H).

## DISCUSSION

We described previously the potential of adenoviral therapy using the replicative *E1B*-attenuated ONYX-015 (dl1520, CI-1042) in human malignant glioma models *in vivo* (Geoerger *et al*, 2002). ONYX-015 displayed significant antitumour activity in *p53* mutant IGRG88 and in *p53* wildtype IGRG93 and IGRG121 advanced stage tumours. Interestingly, our wildtype *p53* gene sequence tumours were more susceptible than the *p53* mutant tumours experiencing high rates of tumour regressions and tumour-free survivors. A phase I clinical trial in recurrent malignant glioma (NABTT-9701) is currently ongoing. Nevertheless, it is unlikely that adenoviral therapy applied as a monotherapy will cure glioblastoma. Data from preclinical studies and clinical trials have suggested that ONYX-015 sensitises tumour cells to killing by chemotherapy (Heise *et al*, 1997;^2000^; Vollmer *et al*, 1999; You *et al*, 2000) or radiation therapy (Rogulski *et al*, 2000). A phase II trial of a combination of intratumoral ONYX-015 injection with cisplatin and 5-fluorouracil in patients with recurrent squamous cell cancer of the head and neck showed substantial objective responses (Khuri *et al*, 2000). The mechanisms of interaction between chemotherapy or ionising irradiation and adenoviral therapy are not fully elucidated. In addition, the role of p53 in combination therapy with ONYX-015 has remained largely unresolved. In combination with cisplatin-based chemotherapy, ONYX-015 has shown additive or potentially synergistic efficacy *in vitro* and in human tumour xenograft model studies in p53-deficient as well as in p53-functional tumour cells (Heise *et al*, 1997). In addition, Vollmer *et al*. (1999) suggested a more viral dose-dependent rather than a p53-selective effect of synergistic antitumour activity in hepatocellular carcinoma models. In contrast, You *et al*. (2000) found that the synergistic effect of ONYX-015 and paclitaxel and cisplatin in lung cancer cells was *p53* mutant dependent, and Rogulski *et al*. (2000) and colleagues showed that additive effects of radiation and ONYX-015 in human colon carcinoma were limited to ONYX-015-sensitive, p53-deficient tumours. Radiation therapy following surgical resection is the current first-line treatment strategy for malignant gliomas. The ability of tumour cells to develop resistance to radiotherapy is, however, a principal limitation of the current standard treatment. As viral therapy and radiotherapy act by different mechanisms, it is not unexpected that cross-resistance mechanisms have not been identified so far. For these reasons, combination treatment consisting of radiation therapy and ONYX-015 administration holds promise for glioblastoma. Here we demonstrate additive, even potentiating antitumour activity of ONYX-015 in combination with radiation therapy in two malignant glioma xenograft models *in vivo*. Both tumours exhibited similar and moderate sensitivities to 5 Gy irradiation. In the *p53*-mutant IGRG88, total body irradiation prior to ONYX-015 treatment showed additive tumour growth delay compared to each treatment strategy alone. In the ONYX-015 highly sensitive, p53 functional IGRG121, concomitant therapy at the same dose of irradiation and ONYX-015 at a 10 times lower virus dose, which, in itself was ‘subtherapeutic’, resulted in potentiated tumour growth delays and tumour-free survivors. To provide some insight into the mechanism of interaction between irradiation and ONYX-015 in glioma, we investigated the effect of radiation on ONYX-015 infection and replication in p53 wildtype and mutant glioma cells *in vitro* and *in vivo*.

Group C adenovirus infection is dependent upon the expression of the primary and secondary receptors CAR and the *α*v integrins on the target cells for binding and internalisation, respectively (Bergelson *et al*, 1997; Tomko *et al*, 1997). Therefore, we first investigated if radiation therapy sensitises tumour cells to adenoviral infection through upregulation of the expression of adenovirus receptors. Without irradiation, CAR was expressed on IGRG88, but not on the highly ONYX-015 sensitive IGRG121 cells (Geoerger *et al*, 2002). Irradiation did not significantly increase expression of adenoviral receptors in malignant glioma cells *in vitro*. In addition, viral infection and gene transfer efficiency using a luciferase adenovirus vector was not increased through irradiation *in vitro* (Figure 2). Thus, irradiation did probably also not enhance ONYX-015 infection *in vivo* by facilitating cell entry.

We next investigated cell-cycle status upon irradiation because it has been described that cells in the S phase are more sensitive to lytic activity of adenovirus than those in the G0/1 phase (Goodrum and Ornelles, 1998). The two tumour xenografts included in this study differed only little in the percentage of cycling cells *per se*. Irradiation induced a transient increase of S-phase fraction of only 3–5% after 7 h, followed by cell-cycle arrest and a subsequent drop of S-phase fraction to 50% of the initial percentage (Figure 3). Although transient increase of cycling cells might facilitate adenoviral infection, the following increase of cells in G0/1 and G2 M during growth arrest could even decrease their sensitivity thereafter. Therefore, we concluded that irradiation did not potentiate ONYX-015 by changing the cell cycle profile.

Next, viral replication of ONYX-015 following irradiation was evaluated *in vivo* by measuring quantitatively viral yields in treated tumours and by immunohistochemistry for adenoviral hexon protein to visualise virus-infected cells in tumour sections. Recently, another replication-competent, oncolytic adenovirus, the prostate cancer-specific CV706, showed substantial synergistic antitumour efficacy in combination with radiation therapy (Chen *et al*, 2001). High-dose irradiation increased viral titres in the human prostate cancer cell line LNCaP *in vitro*, but this was not confirmed *in vivo*. Surprisingly, we observed that tumours treated with irradiation and ONYX-015 contained less virus compared to tumours treated at the same virus dose alone and much less than tumours injected with the 10-times higher dose of ONYX-015. Immunohistochemistry confirmed these findings. We concluded therefore, that irradiation of glioma tumours does not enhance ONYX-015 infection and replication. We scheduled our *in vivo* experiments using 5 Gy TBI followed 6–7 h later with the first virus injection and observed potentiating antitumour efficacy. In both previous reports on combined adenoviral and radiation treatment, however, adenovirus was given prior to irradiation. Also, these studies showed additive or synergistic effects. The efficacy of combined radiotherapy and adenoviral therapy does not seem to be dependent on the sequencing of both agents, but needs to be further explored within the same tumour model.

Although ONYX-015 was administered after irradiation, it is still conceivable that adenovirus replication potentiated radiation-induced processes. ONYX-015 expresses functional E1A proteins. Malignant tumours, when expressing adenovirus E1A, are very sensitive to *in vivo* treatment with DNA-damaging agents, including irradiation (Sanchez-Prieto *et al*, 1995, 1996; 1998; Martin-Duque *et al*, 1999). In addition, sensitisation of p53-functional tumour cells may involve induction of high levels of p53 protein by E1A expression (Lowe and Ruley, 1993). Indeed, intratumoral ONYX-015 treatment induced significant expression and stabilisation of the p53 protein in the p53 wild-type IGRG121 tumour (Geoerger *et al*, 2002). ONYX-015 replication might therefore induce p53-dependent growth arrest and/or cell death, additional to radiation-induced effects. The absence activation of p53 could be one reason for the less evident effect of the combined treatment in our p53 mutant IGRG88 model. However, there are many potential differences between tumour cells beside a varying p53 gene sequence, and a difference in their sensitivity may only be attributed to p53 unless evaluated in otherwise perfectly matched cell lines. Moreover, the infectability of p53 wildtype glioma cells even raises concerns about the implications of this adenovirus on normal brain tissue. Nevertheless, tumour cells with functional p53 are still not ‘normal’ cells. Quiescent or slow proliferating cells do not sustain the replication of ONYX-015, however, infection cannot be completely excluded to current knowledge. Nonattenuated adenoviruses replicate inefficiently and undergo an abortive infection in mouse and rat tissue cells. Thus, rodent animal models are not adequate settings to test toxicity of replication-competent adenoviruses. Definite proof of safety and efficacy will unfortunately have to await results from clinical trials with ONYX-015. To date, no significant toxicity has been reported following intratumoral, intraperitoneal, or intrahepatic arterial injections into cancer patients in Phase I and II trials.

IGRG121 tumours treated with the high dose of ONYX-015 (5 × 10^8^ PFU) showed major post-therapeutic changes, including apoptosis, single cell necrosis and stromal fibrosis. However, these changes were not seen in tumours treated with radiation alone or with radiation and ONYX-015 at the lower dose. In the latter case, antitumoral effects were primarily associated with infiltrations of macrophages and lymphocytes, and predominant, early appearing tumour fibrosis. We therefore suggest, that the potentiating antitumour activity of radiation therapy and ONYX-015 is related to an induction of inflammatory processes through adenoviral infection and subsequent acceleration of radiation-induced processes of tissue fibrosis.

In summary, our investigations demonstrate the potentiating antitumour efficiency of local treatment with the *E1B*-deleted, replication-competent adenovirus ONYX-015 in combination with radiation therapy in human glioblastoma xenografts. The combination treatment was particularly effective on the p53 functional IGRG121 xenografts. Although it remains to be determined whether ONYX-015 exerts any toxicity to normal brain tissues, combination therapy with replication-selective oncolytic viruses and radiation therapy holds promise as a new treatment paradigm for glioma.
